# Measurement of Protein Transport in Heterogeneous Environments Using Confinement k-Space Image Correlation Spectroscopy

**DOI:** 10.3390/biom16040519

**Published:** 2026-03-31

**Authors:** Elvis Pandžić, John W. Hanrahan, Asmahan Abu-Arish, Paul W. Wiseman

**Affiliations:** 1 Katharina Gaus Light Microscopy Facility, Mark Wainwright Analytical Center, UNSW Sydney, Sydney, NSW 2052, Australia; e.pandzic@unsw.edu.au; 2 Department of Physiology, McGill University, Montreal, QC H0H H9Z, Canada; john.hanrahan@mcgill.ca; 3 Department of Anatomy, Physiology and Pharmacology, University of Seskatchewan, Saskatoon, SK S7N 5E5, Canada; 4 Physics and Chemistry Departments, McGill University, Montreal, QC H3A 0C8, Canada

**Keywords:** k-space image correlation spectroscopy, confinment diffusion, membrane domains, GPI-anchored proteins

## Abstract

Current models of the cell membrane assume a heterogenous environment such as sphingomyelin and cholesterol-enriched nano- and microdomains, which are thought to functionally sequester proteins. Besides lipid-ordered domains, membrane proteins can interact with protein complexes by transient binding—necessary for their functional role. Here, we show that an extension of k-space Image Correlation Spectroscopy applied to standard fluorescence microscopy image time series can be used to characterize the protein confinement in heterogeneous membranes. To validate this method, we simulated confined diffusion of tracer particles in a system of static microdomains where we varied the domain size, domain density, confinement probability and diffusion coefficients of tracer particles. We show how the kICS correlation function changes with these parameters and gives rise to emergent properties of the system such as apparent domain sizes and characteristic diffusion coefficients. As a validity check, we apply this analysis to study the dynamics of lipid domain-associated glycosylphosphatidyl inositol (GPI)-anchored proteins labeled by green fluorescent proteins (GPI-GFP) in intact COS-7 cell membranes, and upon domain-disrupting enzyme treatments.

## 1. Introduction

Since lipid domains were first hypothesized by Simons and van Meer [1,2], the existence and properties of these cell membrane domains have been investigated by a wide range of biochemical and biophysical techniques, with the goal of clearly defining their size, composition, and lifetime. One of the most studied lipid domain-associated proteins, glycosylphosphatidyl inositol (GPI), was identified by Förster resonance energy transfer (FRET) measurements to exist in small (∼3 nm) cholesterol-dependent clusters [3]. Single-particle tracking (SPT) studies of GPI revealed the existence of mobile small rafts, while larger stationary domains were found to include several proteins [4]. Another report [5] demonstrated that GPI-anchored proteins form transient homo-dimers that are subunits of higher-order oligomers when highly expressed under physiological conditions. Photo-activated localization microscopy (PALM) measurements [6] suggested the existence of protein clusters containing 2–3 GPI proteins, but when applied to COS-7 cells expressing a photo-activable PAGFP-GPI, it revealed a significant number of ∼320 nm diameter clusters. Spot-varying Fluorescence Correlation Spectroscopy (FCS) revealed the partitioning of membrane proteins on time scales of a few μs to ms [7], and a high-spatial-resolution version of same technique, based on stimulated emssion depletion (STED-FCS) [8], revealed a mean domain radius of ∼100 nm. A clever combination of fast imaging via sCMOS camera, combined with similar analysis on per pixel basis, allowed researchers to investigate the diffusion of lipids in range of 0.5–2.5 μm^2^s^−1^, with this approach named Imaging Total Internal Reflection Fluorescence Correlation Spectroscopy (ITIR-FCS) [9]. All of these data suggest that membrane protein-sequestering lipid domains might exist in cell membranes, with a range of spatial sizes and lifetimes spanning several orders of magnitude.

The imaging-based fluorescence fluctuation method, Image Correlation Spectroscopy (ICS), has also been applied to study the diffusion and confinement of membrane proteins. For instance, the Cystic Fibrosis Transmembrane Conductance Regulator (CFTR) channel was measured by temporal ICS (TICS) to give mobile fraction and an effective diffusion coefficient [10]. TICS was also used to measure the mobilities of GPI clusters [11] in cells. These studies produced some useful results but were limited by the fact that they only relied on temporal fluctuations from single-pixel stacks in the image series. Extensions of ICS, such as spatio-temporal ICS (STICS) [12] and k-space ICS (kICS) [13,14], take advantage of both spatial and temporal correlations accessible from a fluorescence microscopy image time series. The kICS approach can be used to measure protein transport, independent of the probe. An extension of STICS originally proposed unsing the time evolution of correlation function width to measure local diffusion in cells [15]. Later works fully explored its connection to the diffusion laws extracted from an image time series [16,17]. In this manuscript, we develop and explore a new approach for studying membrane heterogeneity, using an extension of kICS that characterizes the emerging dynamics of membrane protein populations when proteins diffuse in a heterogeneous 2D environment. To differentiate this approach from standard kICS, we will refer to this extension as confinement k-space Image Correlation Spectroscopy (c-kICS) throughout this manuscript. We already applied c-kICS to study and compare the confinement of wild-type and mutant CFTR in the plasma membrane of primary human bronchial epithelial cells over-expressing EGFP-CFTR [18,19,20]. Moreover, c-kICS was applied to study cholesterol-dependent function and confinement dynamics changes for the human Piezo1 channel in situ [21]. Furthermore, c-kICS was able to follow changes in membrane diffusion and confinement of the death receptor (DR5) mediated by the microtubule cytoskeleton, which plays a role in pancreatic cancer cell death [22]. More recently, c-kICS’s capacity as a complementary tool to super-resolution microscopy was tested in studies on clustering and confinement dynamics of the linker for the activation of T-cell (LAT) proteins [23]. Despite the above applications, a full exploration of the capabilities and limitations of c-kICS was not presented until now. To validate our approach, c-kICS analysis was applied systematically to computer simulations, where particle diffusion coefficients, probabilities of entering and escaping domains, domain sizes, and densities were varied according to values suggested by previous experimental studies [6,7,8,24,25]. We test this approach with a known canonical GPI-anchored protein that undergoes lipid domain confinement on the plasma membrane of living COS-7 cells. Finally, we include in this manuscript’s Supplementary Materials a detailed manual for the installation and usage of a GUI we developed for performing this type of analysis, and we make it available to the community through our GitHub page.

## 2. Theory

In this manuscript, we are presenting the theoretical framework emerging from k-space Image Correlation Spectroscopy analysis of microscopy image data exhibiting confined motion of proteins in heterogeneous cellular environments. For readers unfamiliar with basic Image Correlation Spectroscopy tools (ICS, TICS, STICS), we would like to suggest the reader to consult a detailed beginners’ guide to ICS approaches, with scripts for simulation and analysis providing learners a complete experience to better assimilate this knowledge through exercises [26].

For the purpose of the current manuscripts’s development, we start with an equation for the k-space time correlation function (CF) for M freely diffusing populations (Equation (30) [13]), which was derived for kICS theory of particles in a homogeneous environment:

(1) $$r\left(k^{2},\tau \right)=\frac{I_{0}^{2}\omega_{0}^{4}\pi^{2}}{4}e^{\frac{-k^{2}\omega_{0}^{2}}{4}}\sum_{p}^{M}N_{p}q_{p}^{2}\langle \Theta_{p}\left(0\right)\Theta_{p}\left(\tau \right)\rangle e^{-k^{2}D_{p}\tau }$$

where q_*p*_ is a constant that takes into account the quantum yield of the fluorophore, the collection efficiency, and the detector gain for particle population p. N_*p*_ denotes the total number of particles of species p in the image. D_*p*_ is the diffusion coefficient of species p. $I_{0}$ denotes the laser intensity at the center of the focus, and $\omega_{0}$ is the $e^{-2}$ of the focal volume’s beam radius in the lateral direction. The third spatial dimension was not included, as the measurements are made on flat quasi-2D membranes of thickness more than an order of magnitude smaller than the axial resolution. $\Theta_{p}\left(t\right)$ is a function representing the photo-physics (photo-bleaching, emission blinking, etc.) of species p and equals 1 when a particle is emitting at time t and is 0 when not emitting a particle. For negligible photo-bleaching or in cases of blinking with a rate faster than the imaging frame rate, we will ignore the contribution from the photo-physics temporal CF inside the summation. In the above expression, we also ignored, without loss of generality, the directed transport (flow) of any species p. The full expression, including the flow contribution, can be found in the original derivation of kICS theory [13,27]. For each diffusing particle population, p, the correlation amplitude reflects the fractional contribution of that population as weighted by number and squared yield. In the case of M = 1, we obtain the original kICS result for a single freely diffusing species:

(2) $$r\left(k^{2},\tau \right)=N\frac{q^{2}I_{0}^{2}\omega_{0}^{4}\pi^{2}}{4}\langle \Theta \left(t\right)\Theta \left(t+\tau \right)\rangle e^{-k^{2}\left(D\tau +\frac{\omega_{0}^{2}}{4}\right)}.$$

**Figure 1:**
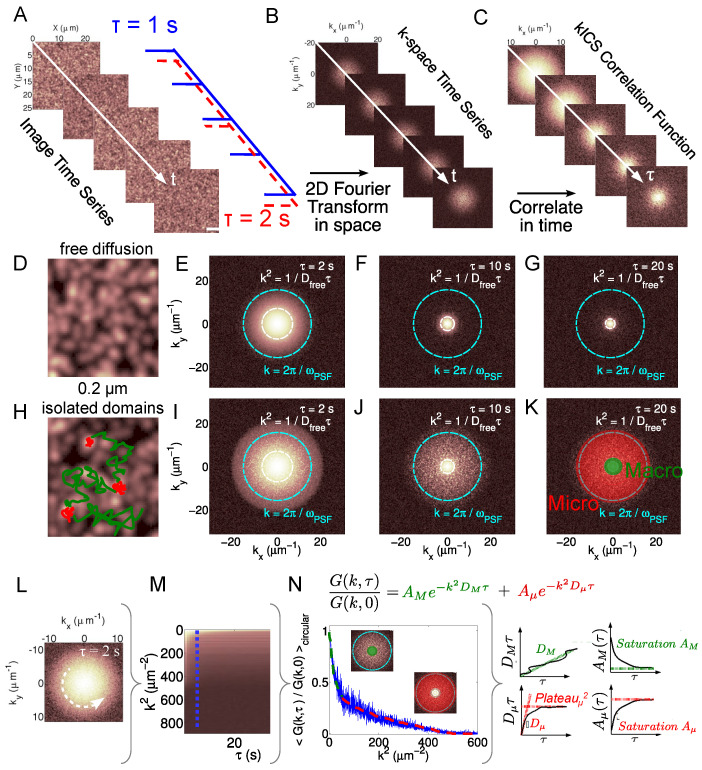
(**A**–**C**) Schematic showing kICS calculation pipeline for a simulated case of a single species diffusing freely with D = 0.1 μm^2^/s imaged with a time frame of 1 s. (**A**) A simulated image series in time. (**B**) Image series Fourier transformed into k-space. (**C**) Temporal correlation function in k-space. Scale bar: 5 μm. (**D**) depicts simulated free 2D diffusion for a single species, and (**E**–**G**) shows kICS CF density plot outputs at different time lags (D = 0.01 $\frac{\mu m^{2}}{s}$, $\omega_{0}$ = 0.4 μm, t_*frame*_ = 1 s). H depicts the simulation of a diffusion on a 2D surface containing randomly distributed circular domains (r_*domain*_ = 0.2 μm, domain area coverage 5 %, D_*in*_ and D_*out*_ set to 0.005 and 0.01 $\frac{\mu m^{2}}{s}$, while P_*in*_ and P_*out*_ set at 0.5 and 0.1, respectively). (**D**,**H**) show schematics of sample particle trajectories in free and confined cases, respectively, superimposed on snapshots from the simulated time series. (**I**–**K**) shows kICS CF density plot outputs at different time lags, for simulated confined case scenario. The red and green areas superimposed on the correlation function in (**K**) highlight the relevant k-vector regimes at which the two dynamic populations emerge. These are labeled micro (μ) for particles diffusing within the domains (red in (**H**,**K**)) and macro (**M**) for particles freely diffusing outside of the domains (green in (**H**,**K**)). (**L**) The kICS CF is circularly averaged for each time lag τ. (**M**) The dashed blue line and its amplitude profile (**N**) show the CF at τ = 5 s. (**N**) Each CF obtained is fitted vs. k^2^, for every τ, with a sum of Gaussians to account for the emergent macro (green) and micro (red) dynamical components. The amplitudes and exponents are functions of temporal lag and are characterized by linear fitting over different temporal lag regimes, in order to extract characteristic c-kICS parameters.

For single-species kICS, the natural logarithm of r is linearly fit vs. k^2^ at a given temporal lag τ. This yields a slope value ($D\tau +\frac{\omega_{0}^{2}}{4}$) at every τ. Plotting the slopes as a function of τ and linear fit gives a slope, which is proportional to the diffusion coefficient of the freely diffusing population. An example of kICS CF for freely diffusing molecular species is shown in Figure 1C–G.

In the case of two freely diffusing species (M = 2), the zero temporal lag r$\left(k^{2},0\right)$-normalized correlation function becomes

(3) $$\frac{r(k^{2},\tau )}{r(k^{2},0)}=\frac{N_{1}e^{-k^{2}D_{1}\tau }+N_{2}e^{-k^{2}D_{2}\tau }}{N_{1}+N_{2}}$$

where the normalized number densities of two species, $N_{i}$, are assumed to be constant in space and time over the measurement interval. In the application reported here, there is a single species with two different dynamic properties (confined and unconfined diffusion), so we assume the quantum yields of the two dynamic populations are equal, which is $q_{1}=q_{2}$ in this case. Photo-physics such as bleaching or emission blinking of particles will naturally affect the amplitudes $N_{i}$, but the effect is assumed to be the same for both dynamic populations so the two amplitudes would be systematically changed by the same ratio. Fitting a sum of Gaussians per Equation (3) to the kICS CF will lead to both of the exponents D_1_τ and D_2_τ being linear with τ. This can be demonstrated by a simple simulation of two populations of freely diffusing particles (see Figure S1).

In the case of two inter-converting species, such as particles freely diffusing for a fraction of the time and being confined (or trapped) at other times, the CF will also effectively be a sum of two independent exponential decays as a function of $k^{2}$.

Nevertheless, the amplitudes and exponents of this CF will depend on the spatial frequency (k) and the time lag (τ) in a non-trivial way as well as on the kinetic parameters defining the inter-conversion rates:

(4) $$r\left(k^{2},\tau \right)=f_{1}\left(k^{2},\tau \right)e^{-g_{1}\left(k^{2},\tau \right)}+f_{2}\left(k^{2},\tau \right)e^{-g_{2}\left(k^{2},\tau \right)}.$$

The fitting parameters *f*_1_ and *f*_2_ are no longer constants of space and time and the exponents *g*_1_ and *g*_2_ are no longer a simple linear relation with time lag τ as in the case of two freely diffusing species (Equation (3)). Moreover, the amplitudes and exponents of the CF will depend on the system kinetic parameters governing the inter-conversion of particles from a free to confined state and back. A feature of the kICS analysis of the temporal image series of particles diffusing in heterogeneous environment is the lack of correlation decay at later time lags on certain l-vector scales due to an effective confined contribution to the CF. In c-kICS, we label this the ‘micro’-scale population, while the particles diffusing freely on longer spatial scales, between the episodes of confinements, is labeled the ‘macro’-scale population (Figure 1K). The fitting of the c-kICS correlation function by Equation (4) leads to diffusion coefficients and relative number densities of these emerging populations, which can be used to study membrane protein confinement by either lipid domains or membrane complexes. The full theoretical derivation of this trapping model, which leads to the sum of Gaussians, was published earlier [28]. Meanwhile, an equivalent theory for the 2D chemical reaction–diffusion model for kICS was also published in [29].

## 3. Materials and Methods

### 3.1. Cell Culture, Protein Labeling, and Enzymatic Treatments

GPI-GFP experiments were conducted in COS-7 cells, which have been extensively used in previous cellular studies of lipid rafts and GPI partitioning into membrane lipid domains [6,7,25,30,31]. The COS-7 cells were cultured and passaged according to standard procedures prescribed for this cell line. Briefly, cells were maintained at 37 °C 5 % CO_2_ incubator in glucose (0.45% *w*/*v*), sodium-pyruvate (0.15% *w*/*v*), and l-glutamine (4 mM)-enriched Dulbecco’s Modified Eagle Medium (DMEM), supplemented with 10% fetal bovine serum (FBS). Confluent cells were passaged (diluted) every 3 days. Five days before imaging, cells were seeded onto glass-bottom (glass N° 1.5 of thickness 0.16–0.19 mm) MatTek (MatTek Corporation, Ashland, MA, USA) dishes. Cells were transfected with a GPI-GFP construct using a Lipofectamine 2000 kit (Invitrogen, Carlsbad, CA, USA). The growth medium was replaced with a nutrient-reduced medium (Opti-Mem I, Invitrogen) 24 h prior to imaging to induce gentle starvation for metabolic consistency. Before imaging, the medium was changed from Opti-Mem I to Hank’s Balanced Salt Solution (HBSS) supplemented with 10 mM HEPES buffer. Cells were imaged before and after enzymatic treatments with either cholesterol oxidase (COase: Sigma Aldrich, Cat. S8633) or sphingomyelinase (SMase: Sigma Aldrich, Cat. C-5421). Enzymes were added to a final concentration of 0.5 Units/mL for SMase and 1–2 Units/mL for COase. Cells were incubated with either enzyme for either 1 h (long exposure) or 15 min (short exposure) at 37 °C prior to imaging. GPI-GFP was labeled with an anti-GFP IgG antibody conjugated with Alexa-594 fluorophores (Life Technologies, Cat. A-21312). For GPI-GFP labeling, 2 μL of the fluorescent antibody was added (0.2 mg/mL) to 1 mL of the cell solution for 15 min, followed by cell washing with HBSS/HEPES buffer twice. A brief exposure of cells to the dye ensured sufficient surface labeling of GPI-GFP by antibodies without extensive internalization. Labeled cells were imaged immediately (control measurements) or treated with enzymes, as explained above, and then imaged.

### 3.2. TIRF Microscopy

Fluorescence microscopy imaging was performed using a TIRF III Research platform (Carl Zeiss, Oberkochen, Germany) on an Axio Observer Z1 microscope, using a 100X Alpha-Plan APO oil immersion objective lens (NA = 1.46), equipped with a cooled electron-multiplying charge-coupled device camera (Evolve S12 EMCCD, Evolve, Denver, CO, USA) of 512 by 512 pixels area. The exposure time of an image was set to 30 ms and, when added with the registering time, gave a total image frame time of 47 ms. Solid-state laser lines of 488 nm and 561 nm with 100 and 40 mW output power, respectively, were used to excite GFP and Alexa 594, respectively. The data were acquired using the AxioVert software customized for this microscope. Each image series consisted of 2000 total image frames with an area of 256 by 256 pixels (25.6 × 25.6 μm^2^. For each condition (control and enzyme treatments), 20 image series were acquired sequentially.

Optics were set such that the pixel size was 0.1 μm. The microscope stage was equipped with an enclosed heating module (37 °C). Beam collimation and the critical angle calibrations were performed as described in the Zeiss user manual. The calibration procedures were performed with tetraspeck fluorescent beads (TetraSpeck Microspheres, Thermo Fisher (Waltham, MA, USA), 0.1 μm radius) diluted in tap water and mounted on a glass-bottom MatTek dish with a thickness appropriate for TIRF microscopy (0.16–0.19 mm). Following calibrations, a cell culture dish was mounted on the stage and coupled by immersion oil to the objective for 15–20 min prior to imaging, ensuring a thermal equilibrium between the cells and the microscope stage.

### 3.3. Computer Simulations

All simulations and data analyses were performed using Matlab 2012a (The Mathworks Inc., Natick, MA, USA). The simulation of Brownian particle diffusion in a 2D environment containing fixed circular domains was carried out as follows: domains were stationary in space of a finite size and randomly distributed uniformly in a region defined by the boundary of an image. The centers were selected to be at least twice the domain radius apart, to avoid potential overlaps. The domain density per μm^2^ was adjusted to preserve the domain fractional area coverage (fixed to 5% unless stated otherwise) in order to account for a change in radius between simulations. Initially, point particles were randomly distributed uniformly within the image frame. Particles were set to move with different diffusion coefficients inside (D_*in*_) and outside (D_*out*_) of domains. When encountering a domain boundary from the inside, a random number from 0 to 1 was generated and compared to the user-fixed probability for a particle to leave the domain, P_*out*_, which could be set from 0 to 1. If the random number was greater than P_*out*_, the particle escapes the domain. Otherwise, it would remain trapped within the domain. Similarly, if a particle encountered a domain boundary from the outside, the user-fixed probability to partition into a domain, P_*in*_ (set range 0–1), was compared to a randomly generated number and an entry decision was made. This simulator does not account for potential gradients in the diffusion coefficients’ contribution to the displacement of particles at the boundaries of domains, as was implemented in recent works [32]. We acknowledge that this contribution could serve as the next iteration of this work, as well as the effect it could potentially have on kICS CF. The periodic boundary condition was implemented to keep the total number of particles constant. An area of 300 by 300 pixels was simulated and the central 256 by 256 pixels region was used for analysis to model a real imaging experiment where a cell surface ROI was open to membrane protein transport. The pixel size was set to 0.1 μm. D_*in*_ and D_*out*_ were set to 0.001 and 0.01 $\frac{\mu m^{2}}{s}$, respectively, unless otherwise stated. The frame time was adapted to accommodate the size of domains as $t_{frame}=\frac{{\left(\frac{\mathrm{domain}\;\mathrm{radius}}{5}\right)}^{2}}{4D_{in}}$. The number of simulated frames was set to 2200, and an adjustment of the time frame allowed for sampling of a reasonable number of fluctuations inside and outside the domains. The first 200 frames of the image series were not included in the calculation of the correlation function, to allow the particles to reach a partitioning steady state, for the confinement scenarios considered. Particle density was set to 5 particles per μm^2^. Photo-physics effects (photo-bleaching and emission blinking) of fluorophores were not simulated for the particles as these should not significantly impact the measured dynamics of particles on the time scales considered. The quantum yield of the particles was set to 1. Background noise was implemented as described previously [33] and varied from 0 to 0.2 as described in Table S1, which summarizes the set of parameters used in the computer simulations. For example, the set background noise parameter at 0.2 reflects the signal-to-noise $\frac{S}{N}$ ratio of $\frac{1}{0.2}$ = 5. Images were constituted by 2D convolution of a 2D Gaussian filter, modeling the PSF of a fluorescence microscope, and are separated in time at a defined frame rate. In order to create realistic images from these sub-pixel particle trajectories, a summation of Gaussian functions placed at the exact coordinates of each particle was made at a given time. Mathematically, this defines a pixel intensity as follows:

(5) $$I\left(i,j,t\right)=I_{0}\sum_{n=1}^{N}e^{\frac{-{\left(i-x_{n}\left(t\right)\right)}^{2}}{2\omega_{x}^{2}}}\cdot e^{\frac{-{\left(j-y_{n}\left(t\right)\right)}^{2}}{2\omega_{y}^{2}}}$$

where $I_{0}$ is the intensity of the Gaussian PSF at the origin, i and j denote integer value coordinates of the pixel, for N particles, $x_{n}\left(t\right)$ and $y_{n}\left(t\right)$ are the sub-pixel coordinates of particle n at time t. This models the integration of fluorescence emission by CCD-type detectors used in TIRF microscopy. The PSF lateral e^−2^ radii $\omega_{x}$ and $\omega_{y}$ in x and y dimensions, respectively, are usually approximately equal for a well-aligned microscope. Therefore, we will refer to them as $\omega_{0}$. For more information about the simulation parameters values used, we would like to refer the reader to Table S1.

### 3.4. Data Analysis

Data analysis was performed in Matlab (The Mathworks Inc, Natick, MA, USA) and a custom GUI for c-kICS analysis was developed and is available, with a guide on installation and usage found at GitHub page https://github.com/ElvPan/Confinement-kICS (accessed on 22 March 2026). Figure 1 gives a schematic overview of the imaging and c-kICS analysis steps, starting from the input image time-series data (Figure 1A) to the calculation (Figure 1L) and fitting of normalized correlation functions, (Figure 1N) to the retrieval of characteristic transport and confinement parameters of the two dynamic particle populations sampled in the image series (confined and freely diffusing, Figure 1N). From early temporal lag fits of the kICS CF, we retrieve the diffusion of molecules at large spatial scales or macro component (D_*M*_), which is related to the overall mobility of molecules throughout the cell. From the fit of the early temporal lags of the micro component, we extract the diffusion coefficient at small spatial scales (D_*μ*_) corresponding to confined mobility of molecules. Also, from the late temporal lags of the micro component plateau, we extract the maximum displacement at small spatial scales (Plateau_*μ*_), which is related to the average domain size. Finally, the late temporal lags of the amplitudes of the correlation functions (Saturations A_*μ*_ and A_*M*_), are related to the number densities of molecules in each dynamical population. Details on the extraction of these confinement parameters can be found in Section 3, while eschematics are found in Figure 1N and examples provided in Figure S4. Furthermore, details on the kICS correlation function computation, fitting, and characterization can be found in the Supplementary Materials.

## 4. Results

**Figure 2:**
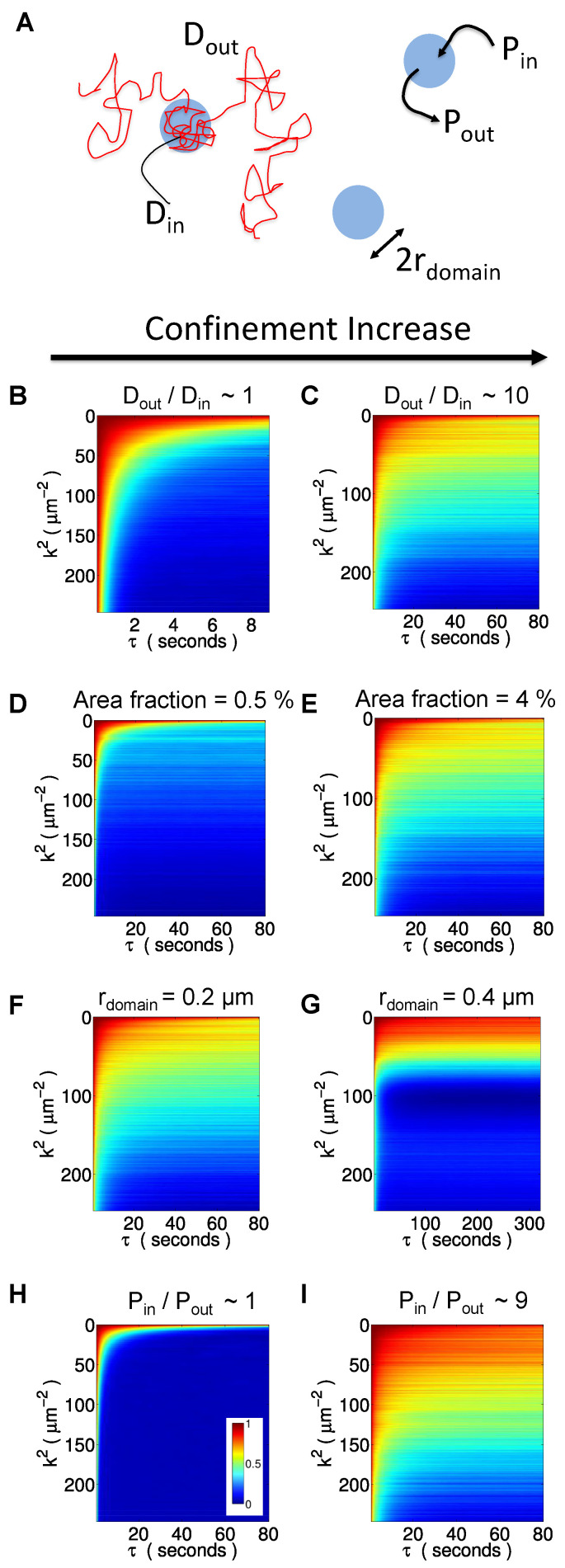
kICS correlation functions for simulated cases with varying confinement parameters. (**A**) Schematic showing what different physical parameters of membrane domains influence the confinement of membrane proteins. Area fraction is the percentage of membrane area covered by the domains. The confinement strength increases as $\frac{D_{out}}{D_{in}}$, area fraction, r_*domain*_, and $\frac{P_{in}}{P_{out}}$ increase. (**B**,**C**): Examples of kICS CF for two scenarios of diffusion coefficients ratio, $\frac{D_{out}}{D_{in}}$= 1 and 10. (**D**,**E**): Examples of kICS CF for simulations with two different values of domain area fraction, 0.5 and 4 %. (**F**,**G**): kICS CF for two simulated cases where domain radii were set to 0.2 and 0.4 μm, respectively. (**H**,**I**): kICS CF for simulated cases of two different probabilities ratio for proteins to enter and escape domains, $\frac{P_{in}}{P_{out}}$ = 1 and 9, respectively. In all kICS CFs, highest amplitude (red) is equal to 1 and lowest (blue) is equal to 0.

The next five sections detail the c-kICS analysis on the simulated image data (see Figure 2A for schematics) of the 2D diffusion of molecules in the presence of confining domains. The domains are simulated as disks with radius r_*domain*_, with defined surface coverage fraction, where molecules diffuse with different diffusion coefficients inside and outside the domains, D_*in*_ and D_*out*_, respectively, and can partition in and out of domains with finite probabilities, P_*in*_ and P_*out*_, respectively. Unless otherwise specified, the domain radius was kept constant at 0.2 μm, and the domain area fraction at 5%, while D_*in*_ and D_*out*_ were respectively set at 0.001 and 0.01, and transition probabilities P_*in*_ and P_*out*_ at 0.5 and 0.1, respectively. Table S1 summarizes the simulation parameters used in this work.

### 4.1. Varying the Diffusion Coefficient Inside the Domains

**Figure 3:**
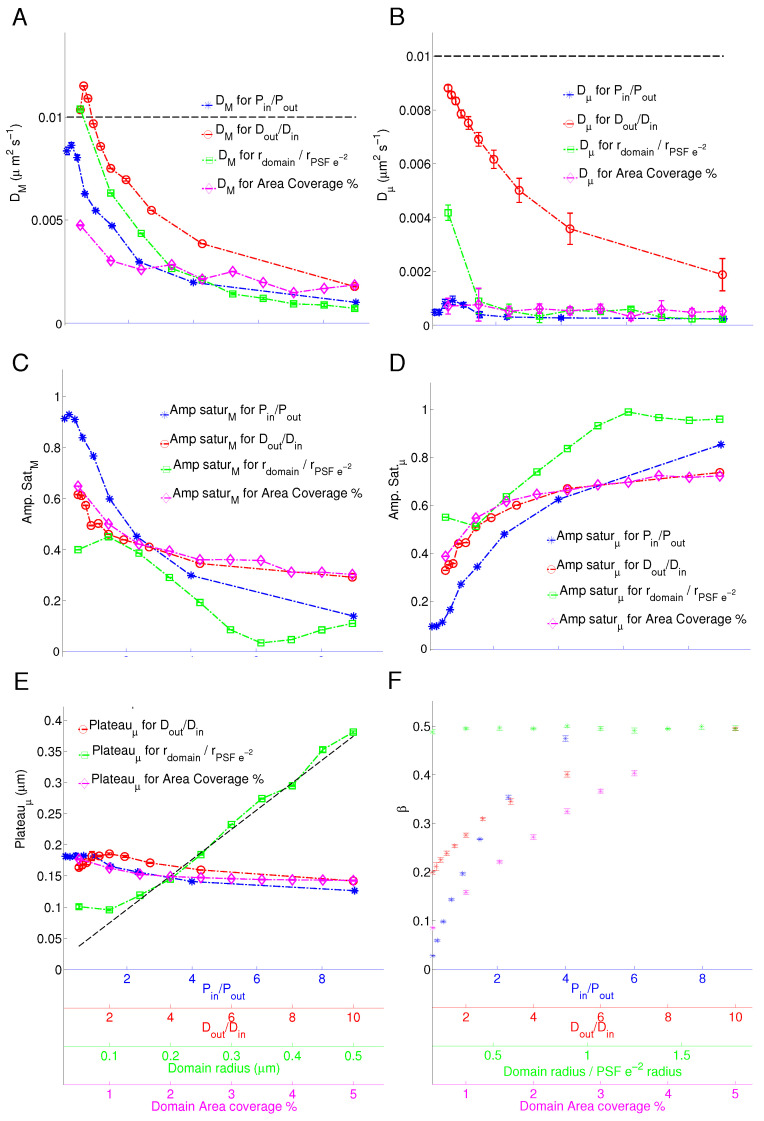
c-kICS analysis confinement parameters (D_*M*_, D_*μ*_, Amp.Sat._*M*_, Amp.Sat._*μ*_, Plateau_*μ*_) extracted for all simulations tested in this manuscript. Red symbols label varying diffusion coefficients inside domains (or change of $\frac{D_{in}}{D_{out}}$) where D_*out*_ (dashed line in (**A**,**B**)) was kept at 0.01 μm^2^s^−1^. Blue symbols show simulations for varying probabilities of molecules going in and out of domains, $\frac{P_{in}}{P_{out}}$, where P_*in*_ varied from 0 to 1 at increment of 0.1, and P_*out*_ was set to 1-P_*in*_. Green symbols show simulation results for varying domain radii from 0.05 to 0.5 μm and keeping PSF x–y plane e^−2^ radius at 0.28 μm. Magenta symbols show results of simulations where the domain area fraction varied between 0.5 and 5 % of the total simulation area. (**A**) c-kICS parameter representing the diffusion of molecules on the macro scale; D_*M*_ (**B**) c-kICS parameter representing the diffusion of molecules on the micro scale, D_*μ*_. (**C**) c-kICS parameter named Amp.Sat._*M*_, measuring the saturation of amplitude at large τs for the macro component. (**D**) c-kICS parameter named Amp.Sat._*μ*_, measuring the saturation of amplitude at large τs for the micro component. (**E**) c-kICS parameter named Plateau_*μ*_, measuring the level at which the square root of the D$\tau_{\mu }$ in the micro component saturates at large τs. Dashed line indicates the linear correlation between Plateau_*μ*_ and simulated domains radius. (**F**) The average partition coefficient β calculated from particles trajectories for varying simulation scenarios considered in this manuscript. Unless otherwise specified, all other simulation parameters were set according to the values detailed in Table S1. Error bars represent the 95% confidence intervals on plotted parameters.

Figure 2B,C show the average correlation functions for simulations in which the diffusion coefficient inside the domain, D_*in*_, varied from 0.001 to 0.01 while keeping D_*out*_ at 0.01 μm^2^/s. Qualitatively, as we decrease D_*in*_, the correlation functions amplitude increases at higher spatial frequencies (k^2^). Indeed, at D_*in*_=0.01 μm^2^/s, the correlation function is almost zero in the range of k^2^ from 100 to 200 μm^−2^, for τ values above 4 s (Figure 2B). As we decrease D_*in*_ to 0.001 μm^2^/s (Figure 2C), that range of the correlation function increases in amplitude. This results from particles spending a longer time inside domains undergoing confined diffusion, where the diffusion coefficient inside the domains is lower. Effectively, a lower diffusion coefficient inside domains results in less frequent encounters with the domain boundary and hence a lower probability for the particle to escape. Red symbols and connecting lines in Figure 3A–E show the c-kICS parameters extracted from fits of the simulations where ratio $\frac{D_{out}}{D_{in}}$ is varied (or D_*in*_ was varied), while D_*out*_ is constant (black dashed line). Figure 3A shows that measured effective D_*M*_ decreases with increasing ratio $\frac{D_{out}}{D_{in}}$. The recovered value is always smaller than the set value of D_*out*_ (black line), so it appears to be an effective diffusion coefficient rather than absolute measurement at small spatial frequencies (i.e., large spatial scales). This indicates that measured D_*M*_ is impacted by D_*in*_. D_*μ*_ also consistently decreases (Figure 3B) with increasing $\frac{D_{out}}{D_{in}}$, as expected. Amplitude saturation of the macro component (Figure 3C), A_*M*_, decreases with increasing $\frac{D_{out}}{D_{in}}$, which implies that with increasing the confinement strength decreases the molecules residing outside domains. Similarly, the increasing $\frac{D_{out}}{D_{in}}$ (implying stronger confinement) increases the number of molecules inside domains, hence an increase in the amplitude of the micro component (Figure 3D), A_*μ*_. The Plateau_*μ*_ extracted from the late temporal lags increases at small $\frac{D_{out}}{D_{in}}$ values, but it then drops continuously (Figure 3E). As $\frac{D_{out}}{D_{in}}$ ratio increases, particles become more confined, which results in them tracing a smaller effective area at small spatial scales. As we decrease that ratio, particles encounter the boundaries of the domains more often, which increases the escape probability. Increasing ratio $\frac{D_{out}}{D_{in}}$ also increases the fraction of trapped particles (β) due to a decrease in their diffusion within domains (Figure 2F).

### 4.2. Varying the Domain Area Fraction

The domain area coverage varied from 0.5 to 5% of the total image area. An increase in the domain area fraction is expected to increase the number of confined particles. Consequently, this results in an increase in kICS CF’s amplitude at higher spatial frequencies at higher temporal lags τ (Figure 2D,E). Note that for all of the correlation functions shown so far have a fast decaying contribution at small spatial frequencies. This is the result of macro population diffusion over large spatial scales. The extracted c-kICS parameters related to the varying domain area fraction are shown in magenta symbols and lines in Figure 3A–E. As the domain area fraction increases, the D_*M*_ decreases as shown in Figure 3A while D_*μ*_ increases slightly (Figure 3B). This could be due to an increase in the fraction of trapped particles (Figure 3F, magenta symbols), hence giving rise to the emergence of the micro-scale diffusing population characteristics in the correlation functions. Increasing the domain area coverage decreases the amplitude saturation A_*M*_ (Figure 3C) while increasing A_*μ*_ (Figure 3D) suggesting an increase in overall particle confinement. Interestingly, the domain area fraction does not affect the recovered apparent domain radius (Figure 3E), except for slight decrease in a Plateau_*μ*_.

### 4.3. Varying the Domain Radius

c-kICS analysis for simulations with variable domain radius resulted in a drastic change in correlation functions, as shown in Figure 2F,G. Increasing the domain radius from 0.2 to 0.4 μm results in lower amplitudes at higher spatial frequencies (high k^2^). The larger the spatial scale explored, the smaller the spatial frequencies visible in the CF. Consequently, the correlation function can be used to infer the effective domain size present in the system. The pixel size in all simulations was set to 0.1 μm and the PSF e^−2^ radius to 0.28 μm. Therefore, at least one simulated scenario contains domains smaller than the pixel size while at least 5 of them have domains smaller or equal to the PSF size.

Inspection of the fitted parameters (green symbols and lines in Figure 3A–E) suggests the recovered D_*M*_ decreases as the domain size increases and is smaller than the set D_*out*_ but remains greater than D_*in*_ (Figure 3A). On the other hand, the early temporal lag fit of D_*μ*_τ vs. τ curve suggests that the mobility of molecules inside domains is lower than D_*in*_ (Figure 3B). An increase in domain radius results in a decrease in saturation A_*M*_ (Figure 3C) while saturation A_*μ*_ (Figure 3D) increases. This is due to an increase in molecule confinement (Figure 3A,B). The value of the Plateau_*μ*_ clearly increases linearly with domain radius squared for domain radii equal to the 0.2 μm or greater (Figure 3E). An interesting observation is that with a theoretical domain radius of zero, the 4× Plateau_*μ*_ value should be around 0.01 μm, which is the size of a pixel squared. This is possibly the smallest spatial scale that could, in theory, produce fluctuations that can be observed from one image to another in a temporal image series. For the fixed $\frac{P_{in}}{P_{out}}$ and $\frac{D_{out}}{D_{in}}$, large domains are expected to contain more particles than small ones since more time, on average, is required by particles to escape domains.

### 4.4. Varying the Probability for Partitioning into Domains

For the variation of P_*in*_, the probability for particles to exit the domain, P_*out*_, was set to 1-P_*in*_. Figure 2H,I show the average correlation functions for simulations in which P_*in*_ and hence $\frac{P_{in}}{P_{out}}$ was varied. The c-kICS analysis for varying $\frac{P_{in}}{P_{out}}$ ratio shows clear trends, as displayed in blue symbols and lines in Figure 3. Both D_*M*_ and D_*μ*_ decrease with the increase in the $\frac{P_{in}}{P_{out}}$ ratio (Figure 3A,B). Indeed, as we approach a ratio of 9, D_*M*_ is reduced to almost the value of the diffusion coefficient inside the domains, while D_*μ*_ approaches zero, suggesting that particles are almost fully trapped. Saturation amplitudes A_*μ*_ and A_*M*_ change dramatically with increasing $\frac{P_{in}}{P_{out}}$. Satuaration amplitude A_*μ*_ increases, as expected (Figure 3D), while saturation A_*M*_ decreases (Figure 3C), suggesting increasing confinement and number of trapped particles with the increasing probability for partitioning into domains. Furthermore, Plateau_*μ*_ (Figure 3E) slightly decreases in value with increasing $\frac{P_{in}}{P_{out}}$ ratio. At intermediate ratios, the Plateau_*μ*_ approaches the value of the domain radius squared, but at the highest ratio, it falls below this value (Figure 3E). This effect is similar to that observed with varying $\frac{D_{out}}{D_{in}}$ (Figure 3E, red symbols).

### 4.5. Characteristic Times for Free and Confined Particles

From the particle coordinates generated in the simulations, the total time a particle spends inside and outside of a domain can be calculated. With that information, the average partition coefficient, β, is calculated to define the ratio of the number of particles within domains at a given time divided by the total number of particles. Therefore, in the limit of β approaching zero, the occupation number of the domains becomes zero. Conversely, β approaching one leads to complete trapping (as in the case of the scenario of $\frac{P_{in}}{P_{out}}=$ 9). Figure 3F shows the values of β calculated from the simulations described in the previous sections. It appears that β and the amplitudes of the micro population exhibit similar trends, except for the case of the domain radius variation. In the case of domain radius variation, total number of simulated domains was increased for smaller domains in order to preserve the 5% domain area fraction. As a result, β does not vary for these simulations, but total density of particles per domain increases with domain size, which would then show green symbols in Figure 3F increasing gradually. It is intuitive, in view of the data presented, that an increasing number of trapped particles would lead to a greater micro component amplitude. The parameter β is often used in spot-varying FCS [25,34] data interpretation. It links the effective diffusion coefficient measured in the presence of domains to that measured in their absence:

(6) $$D_{eff}=D_{free}\left(1-\beta \right).$$

It is interesting to note that the characteristic confinement parameter, D_*M*_, as shown in Figure 3A, follows the trend of D_*eff*_ when considering the values of β calculated. Similarly, the amplitude saturation A_*M*_ follows a trend opposite to β, while saturation A_*μ*_ seems to be proportional to the value of β, for all simulations considered. Therefore, the amplitude saturations of the two dynamic components, macro and micro, extracted from c-kICS analysis are good indicators of the average ratios of particles outside and inside of the domains, respectively.

### 4.6. GPI-Anchored Protein Confinement in COS-7 Membranes

**Figure 4:**
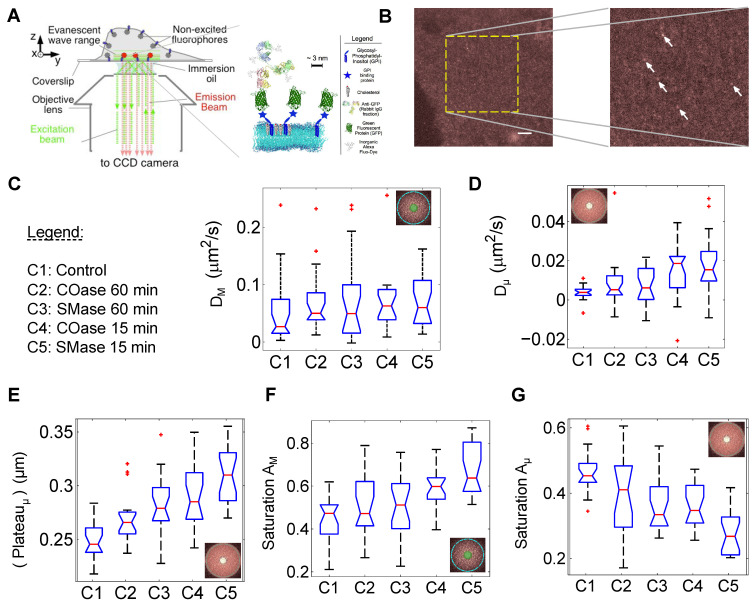
(**A**) TIRF schematic with a zoom-in image of a basal membrane of COS-7, containing GPI-GFP labeled by an anti-GFP tagged with Alexa-594 dyes. (**B**) Example of images acquired with high resolution (512 by 512 pixels) and central 256 by 256 region (yellow square) selected for imaging. Arrows indicate the positions of potential membrane domains. Scale bar in (**C**) is 5 μm. (**C**–**G**) Boxplots for characteristic c-kICS parameters for GPI-GFP extracted from the linear regressions calculated from the correlation function fit parameters for each single COS-7 cell image time series. Red bar indicate the median of measured parameter, box delimit first to third quartile of the data and plus symbols indiacate outliers.

Our first experimental case study is the confinement of GPI-GFP proteins expressed in plasma membrane of COS-7 cells (Figure 4A). To increase GPI-GFP signal-to-background ratio, each GPI-GFP molecule was labeled with an anti-GFP antibody, which has 4–8 Alexa-594 fluorophores (Figure 4A). Addition of 3 μM anti-GFP-Alexa594 into the imaging cell culture dish for 10–15 min labeled enough GPI-GFPs to make them appear brighter against the diffuse background fluorescence of the plasma membrane. This type of labeling is essential to bringing out the fluctuations in single-GPI molecules against the very dense background. Otherwise, we could be biased by motion of bright vesicles or other large clusters enriched in GPI-GFP, as demonstrated in [35]. In the zoomed-in view of the frame shown (Figure 4B), the white arrows label the brighter spots that appear immobile over time, showing fluorescence intensity fluctuations over time. These immobile spots are similar to the isolated domains being populated by GPI-GFP partitioning in and out at defined rates. The fainter features, hidden by the background noise, represent free diffusing GPI-GFP outside of membrane domains.

In order to probe different scenarios of GPI-GFP confinements in the basal membrane of adherent COS-7 cells, samples were exposed to one of the two domain-disrupting enzymes, COase and SMase. These enzymes convert the two major components of membrane domains, cholesterol and sphingomyelin, into new molecules, resulting in an effective domain disruption. They were added to the cells for either long (COase_*long*_ and SMase_*long*_) or short (COase_*short*_ and SMase_*short*_) periods of time, prior to imaging. COase and SMase proved to be most potent within the first hour of treatment. After long periods, these enzymes seem to lose their potency, as cells likely recover some of the converted compounds (cholesterol or sphingomyelin) inside the membrane. COase is known to convert cholesterol into cholestenone and phosphocholine, while SMase converts sphingomyelin to ceramide and hydrogen peroxide. The cholestenone is known to be a potential domain inhibitor due to its different polar head group [36]. Hydrogen peroxide was reported to induce the conversion of the sphingomyelin to ceramide by acidification of membrane bound sphingomyelinase [37]. Accumulated ceramide causes a spontaneous coalescence of domains into larger membrane domains, leading to membrane receptor clustering [19,38]. Ceramide domains are known to be precursors of the cell apoptosis (programmed cell death) [37]. Therefore, the two enzymes employed in these experimental assays can have either raft-disrupting or raft-inducing effects through the various enzymatic reaction products generated. Figure S5A–E shows examples of kICS correlation functions for 5 different conditions. These are the average of correlation functions calculated from ROI time series from 20 different cells (from at least 3 different trials) for each experimental condition. Also, Figure S5F–I presents the average amplitudes and exponents of GPI-GFP dynamics in COS-7 cells as a function of τ under the different treatments. The data presented in Figure S5 show an increase in the mobile population and an increase in D_*M*_τ, while the Plateau_*μ*_ is lower for control samples compared to any of the enzyme treatment conditions.

These two qualitative observations suggest two findings. First, COase and SMase disrupt membrane domains by changing their constituents, which results in a higher mobility of GPI-GFP compared to the control condition, as witnessed by the higher D_*M*_ (Figure 4D). Secondly, the increase in the Plateau_*μ*_, after the enzymatic treatment, suggests an increase in the effective area explored by the confined particles. The amplitude saturations extracted from the non-linear fit of the sum of Gaussians corroborate these qualitative observations. The amplitude saturation A_*M*_ is higher for the enzyme-treated samples than for the control sample (Figure S5G). On the other hand, the amplitude saturation A_*μ*_ is lower for the enzyme-treated samples than for the control sample (Figure S5I). This suggests an increase in the number of particles contributing to the macro component and less to the micro component after the enzymatic treatments. Figure 4 shows the results of the complete c-kICS analysis for this system.

From Figure 4E, it is evident that the median for the Plateau_*μ*_ increases significantly for conditions C1 to C5. (See Figure 4 for legend) Figure 4C shows that the interquartile range (IQR) and the median for all the enzymatic reactions conditions (C2–C5) are shifted upward in comparison to the control sample (C1). The median significance intervals somewhat overlap under control and long SMase (C3) conditions. Nevertheless, there is a clear trend of the increase in median D_*M*_ with the potency of the enzymatic reactions. Interestingly, D_*μ*_ exhibits a significant increase in the median value, especially under short enzymatic exposure conditions C4 and C5 (Figure 4D). Another interesting observation is that all enzymatic conditions (C2–C5) have a larger spread of values and IQR than the control condition. It is possible that after drug treatments, especially for short exposures, GPI-GFP explores small spatial scales at slightly larger diffusion rates than in the control case. The saturation of A_*μ*_ and A_*M*_ show a decrease and an increase, respectively (Figure 4F,G), which is a indicator of decreasing number of confined particles and increasing number of freely diffusing GPI-GFP, as a result of enzymatic treatments.

## 5. Discussion

### 5.1. Simulated Data

A series of simulations were performed, where the set of resulting c-kICS parameters ($\frac{D_{out}}{D_{in}}$, $\frac{P_{in}}{P_{out}}$, r_*domain*_, and $\eta_{d}$) were varied according to values suggested by previous experimental studies, and c-kICS analysis was applied afterwards in order to extract the characteristic confinement parameters. The analysis consists of a calculation of the kICS correlation function from the temporal image series, which is then fit by a sum of Gaussians. The resulting characteristic decays of the correlation function, D_*M*_τ and D_*μ*_τ, as well as their amplitudes, A_*M*_ and A_*μ*_, are a function of time lag, τ. These trends are fit linearly at different τ ranges to extract the confinement parameters such as the diffusion coefficients explored by particles at large and small spatial scales, D_*M*_ and D_*μ*_; the maximum displacement of particles at small spatial scales, Plateau_*μ*_; and amplitudes saturation at high temporal lags, Saturation A_*M*_ and A_*μ*_. When the confinement state increases, the number of particles partitioning into domains increases, which results in a decrease in saturation A_*M*_ and an increase in saturation A_*μ*_. Similarly, one would expect that D_*μ*_ will decrease as particles explore smaller distances at small spatial scales. This is accompanied by a decrease in the Plateau_*μ*_. Moreover, a larger fraction of particles will explore smaller distances at large spatial scales, as they get trapped for longer periods of time within domains, leading to a lower effective diffusion coefficient at large spatial scales, D_*M*_. When ratio $\frac{D_{out}}{D_{in}}$ was increased, it led to a decrease in diffusion coefficient observed outside domains, D_*M*_. Similarly, diffusion at small spatial scales, D_*μ*_ decreased with increasing $\frac{D_{out}}{D_{in}}$. These observations suggest that more particles are confined and have smaller diffusion coefficients at all spatial scales. In agreement with this observation was the increase in saturation A_*μ*_ and the decrease in saturation A_*M*_. Interestingly, the micro component saturation value, Plateau_*μ*_, was increasing up to $\frac{D_{out}}{D_{in}}$∼ 2, followed by a decrease for higher ratios. For ratios of diffusion coefficients smaller than 2, particles diffuse at rates relatively close to the diffusion outside domains, hence encountering the internal boundaries of domains more frequently, which increases their chance of escaping the domains. Consequently, particles explore the area around domains more readily, giving rise to a larger effective measured domain area, or larger Plateau_*μ*_, than the simulation set (domain radius)^2^. On the other hand, when $\frac{D_{out}}{D_{in}}$≥ 2, the lower diffusion coefficient inside the domains increases particle occupancy inside domains, which leads them to tracing the actual domain area. As a result, Plateau_*μ*_ approached the set simulation value of the domain radius.

An increase in simulated domain radius is expected to increase the effective area particles explore at small spatial scales, and this was observed through the linear increase in Plateau_*μ*_ with the increase in domain radius. Similarly, the diffusion coefficient particles explored at small spatial scales, D_*μ*_, measures the effective diffusion that results from particles moving inside and around domains. For smallest domains simulated, with 50 nm radii, the probability of particles encountering domains is relatively low. As result, D_*μ*_ measured is between the diffusion coefficients set for diffusion inside and outside domains, as shown in first green marker of Figure 3B. For larger simulated domains, D_*μ*_ remains relatively constant. This implies that all domain sizes, except those that are very small, the particles will diffuse at same effective diffusion coefficient at small scales, D_*μ*_, as long as $\frac{D_{out}}{D_{in}}$ and $\frac{P_{in}}{P_{out}}$ remain constant. This trend was accompanied by an increase in the saturation A_*μ*_ and a decrease in A_*M*_ with increasing domain radius, suggesting particles are being trapped longer within larger domains. The effective diffusion coefficient at large spatial scales, D_*M*_, decreased with domain radius. This can be explained by the fact that with larger domains, particles are trapped longer from the overall trajectory, so that their effective diffusion coefficient at large spatial scale is smaller.

When we varied the domain area fraction, the macro component mobility and fraction were the most affected confinement parameters. Indeed, D_*M*_ dropped with increasing domain fractional area, which can be explained by particles encountering a larger number of domains as they diffuse on large spatial scales. Similarly, the saturation amplitude of the macro component, A_*M*_, drops with an increase in domain area fraction, which explains that fraction of particles diffusing at large spatial scales is reduced. The saturation of micro component A_*μ*_ increased as the domain area fraction increased, indicating that confinement increases with increasing domain area fraction and with the fraction of confined molecules. These observations are intuitive if one considers that changing the number of domains in the field of view should not affect the partitioning and motion of particles at small spatial scales.

Increasing $\frac{P_{in}}{P_{out}}$ led to a decrease in D_*M*_ and D_*μ*_, suggesting that the higher the probability for particles to enter domains, the lower the effective measured diffusion coefficients on all spatial scales. Again, this is a consequence of particles partitioning more readily into domains with increasing $\frac{P_{in}}{P_{out}}$, which leads to particles traveling shorter distances on large spatial scales (smaller D_*M*_) and results in particles exploring smaller effective distances on small spatial scales, during the same time window (smaller D_*μ*_). As a result, Plateau_*μ*_ approaches the true value of (domain radius)^2^ as we increase $\frac{P_{in}}{P_{out}}$. Saturation A_*μ*_ increases with $\frac{P_{in}}{P_{out}}$ while saturation A_*M*_ follows the opposite trend, as expected.

In view of all of the simulation results, we suggest that different combinations of parameters leading to an increase in the confinement, ($\frac{D_{out}}{D_{in}}$, $\frac{P_{in}}{P_{out}}$, r_*domain*_ and $\eta_{d}$), could generate equivalent confinement states. This idea of degeneracy of confinement states is observed from the statistical parameters (β) presented. Indeed, the same value of the ratio of confined to the total number of particles can be obtained for different sets of $\frac{D_{out}}{D_{in}}$, $\frac{P_{in}}{P_{out}}$, r_*domain*_, and $\eta_{d}$. Similarly, the c-kICS parameters extracted for various simulations scenarios suggest that the degeneracy of confinement states exists. Therefore, it is necessary to perturb the system experimentally, using the domain-disrupting enzymes, in order to shift the system toward one of the equilibrium states and measure the relative change in confinement parameters. Finally, c-kICS parameters reflect apparent diffusion coefficients at micro (D_*μ*_), which can sometimes appear to be lower than the actual diffusion coefficient, as demonstrated via simulations (Figure 3B all but red symbols where set D_*in*_ was 0.001 μm^2^s^−1^). The reason for this apparent observation is that when domains are small, such as set domain radii of 0.2 μm in those simulations, the diffusion inside domains is not free and it is only free within a small spatial range within domains. The fluctuations emerging out of such molecular motions, when biased with collision into inner walls of domains, produce c-kICS CF that when characterized via non-linear fitting of two components will exhibit apparent reduced diffusion coefficient, D_*μ*_.

Lastly, we would like to bring attention to readers on some potential biases that c-kICS could suffer from, as with many other microscopy image data analysis techniques, due to limited optical resolution and imaging noise resulting from various sources in microscopy data collection. We have added Figure S6, which exemplifies how the background noise added to the simulated confinement microscopy image data affects the kICS CF and can consequently affect the reported c-kICS parameters extracted. It is worth outlining that even at a very low signal-to-noise ($\frac{S}{N}$) ratio of 5, kICS CF only becomes corrupt beyond spatial frequencies of k^2^ equal to 60 μm^−2^ or higher. Indeed, the higher spatial frequencies will be mostly affected since noise mostly affects correlation of fluctuations above the 1–2 pixel range. Nevertheless, kICS CF range below 60 μm^−2^ can still be analyzed using c-kICS and extract valuable confinement parameters. A user of c-kICS would have to evaluate the $\frac{S}{N}$ ratio in their data and adjust the upper-bound fitting limit in k^2^ accordingly. Furthermore, a careful experimental design where labeling enhances $\frac{S}{N}$ in image data, as was done in present work or using photo-activable labels [35], will improve the odds in extracting unbiased c-kICS parameters. Moreover, the finite spatial resolution of light microscope, originating from the light diffraction, imposes the limit of smallest resolvable domain we can measure via c-kICS. Inspection of green symbols in Figure 3E shows that for simulated domains of 0.05 and 0.1 μm radii. the c-kICS parameter-extracted Plateau_*μ*_ did not vary linearly. In fact, its value was saturated to 0.1 μm for those small simulated domains. The simulated PSF e^−2^ radius and pixel size were set at 0.28 and 0.1 μm, respectively. Therefore, c-kICS cannot resolve the domains that are smaller or equal to pixels in size and less than half the spatial resolution of microscope, as fluctuations can only occur at the pixel level.

While the present work does not present the applicability of c-kICS analysis in the study of cortico-actin meshwork effect on confinement of membrane-embedded proteins, we would like to invite the reader to consult Chapter 4 (Section 4.9) of the thesis work that inspired this manuscript [39]. Meshwork-based confinement of protein diffusion has been investigated in detail using fast spatio-temporal imaging combined with SPT [40,41] via spot-varying FCS [7] using iMSD analysis [16], with its theoretical framework being well established [42,43].

### 5.2. Experimental Data

In the present study, we report that two effective dynamic populations of GPI-GFP, macro and micro, diffuse at 0.06–0.11 and 0.007–0.02 μm^2^/s, respectively. A similar range of diffusion coefficients for GPI-GFP in COS-7 cells was observed using the UPaint method [44]. This study reports a distribution of GPI-GFP diffusion coefficients to be bimodal, with the peaks of the distribution centered around 0.005 and 0.1 μm^2^/s. The assessment of the GPI-GFP trajectories suggests that confinement zones can be up to ∼500 nm in diameter. The basis of the UPaint technique relies on sparse labeling of GPI-GFP, with anti-GFP antibodies labeled with multiple AT647N dyes. Another approach employed an anti-GFP conjugated with a quantum dot (QD) for the sparse labeling of GPI-GFP [45]. The observed distribution of GPI-GFP diffusion coefficients had peaks at ∼0.25 and 1 μm^2^/s. The larger diffusion coefficient could be attributed to the faster monomeric GPI-GFP. Indeed, similar values for the diffusion coefficient of GPI-GFP were observed by the spot-varying FCS method [25]. Nevertheless, this technique involves an assumption of single dynamic species in their fitting procedure of the FCS’ ACF. Therefore, the single-species model was used in the fit of the temporal ACF, leading to the effective diffusion coefficient of ∼1 μm^2^/s [7]. The same study suggests that GPI-GFP resides within microdomains for 10 to 30 % of the total time, while the average time spent within domains is estimated to be between 34 and 95 ms. The same group applied a two-populations-fit to the FCS ACF and proposed two diffusion coefficients of ∼45 and 0.6 μm^2^/s [24]. In a previous report, they estimated the upper bound of the GPI-GFP microdomain diameter to be ∼120 nm. In agreement with this result is the study [8] of GPI-GFP dynamics by using the FCS-STED, which is an extension of the spot-varying FCS with PSF radius reduced below the diffraction limit, which allows for probing of small spatial scales. STED-FCS estimated GPI-GFP domains to be on the order of 100 nm. Another conclusion from their study was that the COase treatment did not influence the GPI-GFP dynamics. Single-particle tracking has also been applied to follow the movements of Thy-1, a GPI-anchored protein [46]. The results suggested that Thy-1 visits ∼230 nm diameter domains with diffusion coefficients outside the domains in the ranges of 2–8 μm^2^/s, while inside the domains ranges at 1–5 μm^2^/s. This high spatio-temporal resolution measurement of Thy-1 suggests that this protein spends 15% of the total trajectory time inside domains with the average $\tau_{trap}∼$ 5 s. They reported that cyclodextrin depletion of cholesterol decreased the domain size to ∼150 nm in diameter and the protein raft occupancy dropped to 2%, while $\tau_{trap}$ remained unaffected. It is interesting to note that the spot-varying FCS and SPT studies agree only on the estimate of the fraction of time GPI-GFP spends within microdomains. In another report, the translational motion of GPI-linked I-Ek class II MHC membrane proteins in the plasma membrane of CHO cells studied by SPT measured a diffusion coefficient of ∼0.22 μm^2^/s, but it failed to detect any confinement of these proteins [47]. In agrrement with this data, another study [35], using a photo-switchable tagged GPI-mEOS2 expressed in COS-7 membranes, imaged in TIRF mode, and analyzed via STICS and sptPALM, revealed the diffusion coefficients in ∼0.2 μm^2^/s with distribution spanning 0.01 to 10 μm^2^/s. These studies differ from the current study in the sample preparation, as GPI was modifed using a flourescent protein tag rather than antibody labeling, which likely influenced protein mobility. High spatio-temporal resolution SPTs of GPI-anchored proteins demonstrate the existence of transient homo-dimers of GPI [5]. These nano-structures were observed at low GPI-anchored protein expression levels. Nevertheless, the authors suggested that higher surface density of this protein might lead to higher-order oligomers. Other conclusions were drawn about the aggregation state of GPI-GFP as measured by FRET anisotropy [3]. It was demonstrated that ∼30% of GPI-anchored proteins exist in small, ∼3 nm, aggregates composed of up to 4 GPI molecules, while the rest of the GPI were in a monomeric state. Interestingly, the polyclonal antibody cross-linking of GPI-anchored proteins of one species was shown to lead to higher-order oligomers, which was confirmed by the same author in a previous study [48]. In agreement with these results are data from a PALM study that revealed 2–3 proteins per cluster [6]. On the other hand, PALM most frequently detected clusters of PAGFP-GPI with ∼80 nm diameters while larger, ∼320 nm diameter, clusters were also observed.

In light of the literature findings on the dynamics, confinement, and aggregation state of GPI-anchored proteins, we shall summarize the data observed in our current work. The range of diffusion coefficients observed for the large and small spatial scales, D_*M*_ and D_*μ*_, suggest that we are observing a similar dynamic species of GPI-anchored proteins as with the UPaint experiments [44]. A similar diffusion coefficient was observed when GPI was labeled with anti-GFP-QD [45]. In a more recent account [49], an anti-GFP antibody labeled with Atto647N was used to label GFP-GPI and track them in neuronal membranes. They found that diffusion coefficients were less then 0.2 μm^2^/s, but remarkably, authors claimed that the estimated diffusion coefficient can be affected by the length of trajectories recovered in SPT experiments. All of these studies share an important feature with the current study, namely the labeling of GPI-GFP with an antibody. It is possible that anti-GFP-Alexa594 used in the current work clusters 2–4 GPI-GFP together, so that these small clusters form larger domains through sphingolipids and cholesterol interactions. The hypothesis here implies that the large domains observed are a result of the liquid-ordered packing of sphingolipids, cholesterol, and small aggregates of cross-linked GPI. This suggests that monomeric or dimeric GPI-GFP can still partition into and out of these large domains. Consequently, an enzymatic treatment changing sphingomyelin and cholesterol into new products will disrupt the ordered phase, resulting in a faster exchange of particles. The effective area explored at small spatial scales, Plateau_*μ*_, can be traced with a circle of effective radius of 0.250 (control) to 0.310 (short SMase) ± 0.002 μm. It was demonstrated by the analysis of numerical simulations that several parameters can affect this value. The increase in domain size and the decrease in the domain binding rate (P_*in*_) will lead to the increase in Plateau_*μ*_. D_*M*_ doubled in value over the range of enzymatic reactions, going from 0.085 (control) to 0.115 (short SMase) ± 0.002 μm^2^/s. Similarly, D_*μ*_ was measured to be in the range of 0.007 (control) ± 0.007 to 0.02 (short SMase) ±0.02 μm^2^/s. The trend in these three measured confinement parameters suggests that that ratio of $\frac{P_{in}}{P_{out}}$ is decreasing as we go from control to the short enzymatic exposure. The conversion of cholesterol and sphingomyelin into cholestenone and ceramide introduces more permeable domains into the system. Therefore, GPI-GFP will more readily escape these domains, exploring larger distances on both small and large spatial scales. Saturation A_*μ*_ decreased from 0.48 to 0.28 ± 0.01, while saturation A_*M*_ increased from 0.48 to 0.68, indicating again that more GPI-GFP particles explore larger spatial scales following enzymatic treatments. It is important to state that the value of the apparent domain size extracted from the Plateau_*μ*_ should not be taken as an absolute value of the domain size. It was shown through simulations analysis that this value does not depend only on the actual size of domains present. Moreover, the presence of noise in an image series introduces an error in the estimate of this plateau level. Analogous to this is the detection of particle positions in SPT. If the image series’ signal-to-noise ratio is small, the accuracy of the fit particle positions will decrease. Consequently, the MSD curve shifts upwards by a positive value that depends on the variance of the noise present in the image series [46]. As a result, the shifted MSD curve will produce an overestimate of the domain size. Furthermore, the noise can produce an apparent sub-diffusion for the early temporal lags of MSD curves in the SPT data [50]. Nevertheless, the domain sizes reported in the present study are due to several system parameters and, as such, do not represent actual absolute values of domain sizes. Therefore, the image noise effect, depending on its amplitude, will shift the effective domain value by an equal amount for all the image series analyzed. Indeed, both of the control and enzymatic treatment image series were collected under similar conditions of the illumination, time exposure, and EMCCD gain. Hence, the variance of the counting noise is not expected to vary significantly from one series to another. Therefore, relative change is the important aspect of this analysis rather than the absolute value.

## 6. Conclusions

The information extracted from the extensive computer simulations helped in the understanding and interpretation of the experimental results related to the dynamics and confinement of GPI-GFP. The goal of the experiments was to measure the confinement parameters of GPI-GFP–anti-GFP–Alexa594 in live COS-7 cells using the new c-kICS analysis on TIRF-acquired image time series, before and after the treatment with the domain-disrupting drugs, COase and SMase. A longer time exposure to either of these enzymes did not produce very significant changes in GPI-GFP–Alexa594 dynamics and confinement. On the other hand, a shorter term exposure to the enzymes (∼15 min) prior to imaging was more effective in disrupting the sphingomyelin and cholesterol domains. The increase in the diffusion coefficients following the enzymatic reactions, as well as the increase in the saturation of the macro component amplitude and decrease in the saturation of micro component amplitude, suggest that the domains become leaky. The plausible change in the microstructure due to the enzymatic reaction could be that small aggregates of GPI-GFP that are no longer closely packed with sphingolipids or cholesterol are forming. Instead, the newly produced cholestenone or ceramide, depending on the enzymatic reaction, break the ordered and tightly packed microdomain into a more loose domain. The monomers or small aggregates of GPI-GFP can thus explore a larger effective area that is measured with the increase in the Plateau_*μ*_ of the kICS confinement analysis.

As described in the simulation results above, the parameters that affect Plateau_*μ*_ are the domain radii and the ratios $\frac{D_{out}}{D_{in}}$ and $\frac{P_{in}}{P_{out}}$, while the domain area fraction should not affect its value. Also, saturations A_*μ*_ and A_*M*_ change by a factor of two from control to enzymatic treatment conditions. In the simulations with varying $\frac{D_{out}}{D_{in}}$, these amplitude saturations were only obsereved for the range in $\frac{D_{out}}{D_{in}}$ from 0 to 5. For that same range, Plateau_*μ*_ increased up to $\frac{D_{out}}{D_{in}}$∼ 1 followed by a decrease at higher ratios. Together, these suggest that a change in $\frac{D_{out}}{D_{in}}$ is not responsible for a change in confinement parameters following enzymatic treatment of GPI-GFP–Alexa-594 in COS-7, while a $\frac{P_{in}}{P_{out}}$ variation could be occurring.

Image Correlation Spectroscopy (ICS) and various adaptations were previously used to measure protein dynamics in cellular membranes, as well as their aggregation and oligomerization states. kICS was developed to measure protein dynamics, independent of the probe photo-physics. In this work, a new adaptation, c- kICS, was proposed for the measurement of protein confinement and dynamics in a heterogeneous membrane. When c-kICS is applied to an image time series of a system with isolated membrane domains, the correlation function decays effectively as a sum of two exponentials, time lag (τ) and spatial frequency (k^2^). These two decays were linked to two effective dynamic populations that emerge from particles diffusing within the two-phase heterogeneous 2D environment The results of c-kICS analysis of the simulated confinement scenarios suggest that the confinement parameters extracted (D_*M*_, D_*μ*_, Plateau_*μ*_, saturations A_*M*_ and A_*μ*_) correlate with the confinement statistics. Our simulations and experimental verification of GPI-GFP confinment in live COS-7 cell membranes suggest that c-kICS could be used as a quantitative tool for the assessment of the standard confocal and TIRF microscopy images, in search of the confinement state and dynamics of membrane-embedded proteins and receptors.

## Data Availability

An example of data set will be uploaded with this submission. Also, the scripts that generated the simulated data will be available on a GitHub page for downloading. A GUI for c-kICS analysis, with a guide on installation and usage, is available on following GitHub page: https://github.com/ElvPan/Confinement-kICS (accessed on 22 March 2026).

## Supplementary Materials

The following supporting information can be downloaded at https://www.mdpi.com/article/10.3390/biom16040519/s1. Figure S1: kICS analysis of simulations of two freely diffusing populations. Figure S2: Hann windowing is applied to each image prior to the Fourier transform. Figure S3: Non-linear least squares fitting k-space correlation function resulting from a heterogeneous environment. Figure S4: Example of data processing to extract domain size and particle mobilities. Figure S5: Examples of an average correlation functions for GPI-GFP–anti-GFP–Alexa-594 data for 5 enzymatic conditions considered. Figure S6: Examples of c-kICS correlation functions emerging from simulated confinement case, with varying background noise in image data. Table S1: Adjustable parameters for isolated-domain simulations. Video S1: Time-lapse of simulated data with Pin = 0.2 while Pout = 0.1. The frame rate was set to 0.9 s. Video S2: Time-lapse of simulated data with Pin =0.5 while Pout = 0.1. Video S3: Example of a control (no drug treatement) data set of GPI-GFP labeled by anti-GFP–Alexa555 in COS-7 membranes, imaged in TIRF mode at 46 ms per frame. Video S4: Example of a COase-treated sample of GPI-GFP labeled by anti-GFP–Alexa555 in COS-7 membranes, imaged in TIRF mode at 46 ms per frame. Video S5: Example of a SMase-treated sample of GPI-GFP labeled by anti-GFP–Alexa555 in COS-7 membranes, imaged in TIRF mode at 46 ms per frame.

## Abbreviations

CF	Correlation Function
COase	Cholesterol Oxidase
CHO	Chinese Hamster Ovarian cells
CFTR	Cystic Fibrosis Transmembrane conductance Regulator
DMEM	Dulbecco’s Modified Eagle Medium
DR5	Death Receptor 5
EMCCD	Electron-Multiplying Charge-Coupled Device Camera
FBS	Fetal Bovine Serum
FCS	Fluorescence Correlation Spectroscopy
FRET	Förster Resonance Energy Transfer
GFP	Green Fluorescent Protein
GPI	Glycosylphosphatidyl inositol
GUI	Graphical User Interface
HBSS	Hank’s Balanced Salt Solution
ICS	Image Correlation Spectroscopy
IQR	InterQuartile Range
kICS	k-space ICS
c-kICS	confinement-kICS
LAT	Linker for Activation of T-cell
MHC	Major Histocompatibility Complex
QD	Quantum Dot
PAGFP	Photo-Acitvable Green Fluorescent Protein
PALM	Photo-Activated Localization Microscopy
PSF	Point Spread Function
SMase	Sphingomyelinase
SPT	Single-Particle Tracking
STICS	Spatio-Temporal ICS
STED	Stimulated Emission Depletion
TICS	Temporal ICS
TIRF	Total Internal Reflection Fluorescence microscopy
ITIR-FCS	Imaging Total Internal Reflection-FCS
