# Supplementary material for: Measurement of Protein Transport in Heterogeneous Environments Using Confinement k-Space Image Correlation Spectroscopy
Source: Biomolecules. 2026 Mar 31;16(4):519. doi: 10.3390/biom16040519 (PMC13113767; doi:10.3390/biom16040519)
Supplement: Supplementary file 1 [file biomolecules-16-00519-s001.zip › Supplementary Materials.pdf]

Supplementary Materials for *Measurement of protein transport in heterogeneous environments using confinement k-space Image Correlation Spectroscopy*

Elvis Pandžić,John W Hanrahan, Asmahan Abu-Arish, and Paul W. Wiseman

March 20, 2026

kICS analysis for simulations of two freely diffusing populations

Here we explore briefly, the kICS analysis for the case of two freely diffusing populations and demonstrate that the characteristic CF is different from the case of confined d ynamics. D τ trends are linear for both components, unlike the case of confined d ynamics w here o ne o f t he t wo p opulations e xhibit a plateauing. For these simulations no domains were present. The two diffu-sion coefficients se t in th is si mulation, D_1 =0.001 an d D_2 =0.01 $\mu m\ ^2/s$, were the same as D_{in} and D_{out} of the domain simulations described in this work, except that molecules diffused f reely f or t he e ntire s imulated t ime frame. Figure S1 a) shows the average correlation function of 5 trials. There is no significant a mplitude a t l arge k ^{2} v alues f or h igher t emporal l ags, a s i n the confined c ase. M oreover, t he c orrelation f unction d ecays a lmost completely with time. The fit o f t he c orrelation f unction, u sing t he s um o f t wo Gaus-sians as a function of k, produces two straight lines for the characteristic decay constants as plotted Dτ vs τ (Figure S1 b). The macro component fit recovers the larger diffusion c oefficient (D_1) whi le the mic ro com ponent fit recovers the smaller diffusion c oefficient (D_2). Mos t imp ortantly, the micro component of the fit (red circles) does not plateau at long temporal lags (τ). A plateau at long lag times (Pleateau_{μ}) is a hallmark of confinement in the system.

Image series analysis

Image Windowing

The need for image windowing prior to the kICS analysis was previously described (S1). The rationale for the windowing of images comes from the fact that the discrete Fourier transform of an image is sensitive to the sharp discontinuities at the image edges, producing non-existent high frequency components that mix with lower spatial frequencies. We employ the stan-dard procedure to attenuate such spectral leakage by a multiplication of the data with a window function that is equal to 1 at the centre of the signal and decays to zero toward the edges. We used Hann window as it attenuates optimally the spectral leakage in the low spatial frequencies. The 2D ver-sion of the Hann window is made by replicating the existing Matlab function

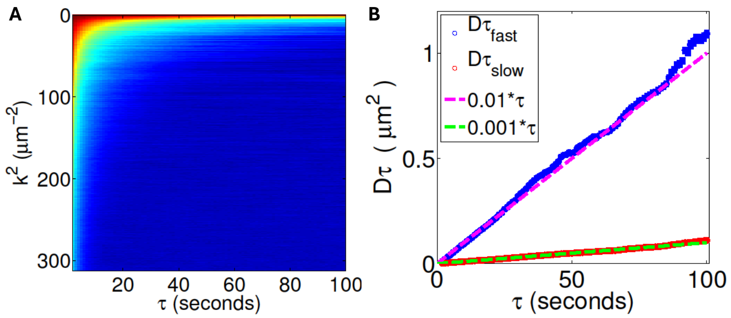

Figure S1: kICS analysis of simulations of two freely diffusing p opulations. (**A**) Amplitude density plot for kICS CF for the case of two freely diffusing populations with coefficients D_1 =0.01 and D_2 =0.001 $\mu m\ ^2/s$. (**B**) Results of the sum of 2 Gaussian fit of the kICS correlation function from (**A**). Blue and red circles denote $D\tau$ from each component of the fit, as a function of τ, while dashed magenta and green lines show linear trends one would get if two freely diffusing species were simulated.

'hann' for 1D signals. The image is multiplied, element-wise, with the win-dow function before the application of the fast Fourier transform algorithm. Figure S2 shows an example of an image before and after windowing and the 2D Hann window function itself.

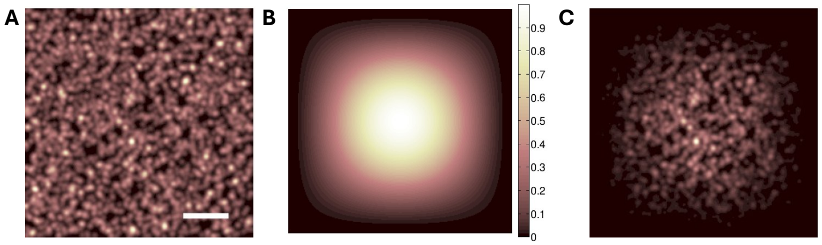

Figure S2: Hann windowing is applied to each image prior to the Fourier transform. (**A**) Simulated image prior to windowing. (**B**) 256 by 256 Hann window. (**C**) Image after windowing. Scalebar is 5 μm

kICS function computation, normalization and averaging

Details of kICS correlation function computation, normalization and averag-ing can be found in (1, 2). Briefly, the windowed images, as described in the previous section, are fast Fourier transformed (by 'fft2' function in Matlab). Then pairs of transformed images, $\tilde{I}(k_x,\ k_y,\ t)$, separated by temporal lag τ, are correlated and averaged producing a non-normalized temporal correlation function:

$$r(k_x, k_y, \tau) = \langle \tilde{I}(k_x, k_y, t)\tilde{I}^*(k_x, k_y, t + \tau)\rangle_\tau \qquad \text{(S1)}$$

The normalization of the above expression with the zero temporal lag CF, eliminates stationary contributions, such as the PSF. The resulting normal-ized correlation function is azimuthally (circularly) spatially averaged, as the system is considered isotropic. This effectively results in the minimum number of fitting parameters, as we get a 1D correlation function. It also reinforces the correlations present at all angles, while averaging out noisier contributions.

Nonlinear fitting of the correlation functions

The correlation functions were fitted using the Matlab function 'fit' from the Curvefit toolbox, with the non-linear least squares fitting scheme. The best results were obtained using the Trust-Region algorithm, as it offers the flexibility for defining the lower and upper bounds of fitting parameters. The bounds were set on the basis that amplitudes of the normalized correlation functions cannot be less then 0 or greater than 1. Similarly, the decay rates of the correlation function's lower and upper bounds were set to 0 and infinity, respectively. The option 'Robust' was used, to ensure that outliers in the data sets are not taken into account during the fit. Similarly, the decay rates were set initially so that one component is an order of magnitude higher than the other. The initial guess of one of the decay rates was estimated by forcing a linear fit through the logarithm of the CF, as done in the standard kICS (2) procedure. This yielded an average diffusion coefficient, that was used as the initial parameter in the non-linear fitting procedure. The ultimate goal was to characterize, through fitting, the circularly averaged and normalized correlation function, $r(k^2,\ \tau)$. Supplementary Fig. 3 shows an example of a calculated kICS correlation function for a simulated confined case. The fitting equation has the form:

$$r(k^2, \tau) = A_M e^{-D_M k^2 \tau} + A_\mu e^{-D_\mu k^2 \tau} + r_{\text{inf}} \qquad \text{(S2)}$$

where A_M and A_μ denote the amplitudes of 'macro' and 'micro' dynamic populations, while D_M and D_μ are the respective diffusion coefficients of the two populations. The value r_{inf} represents the offset of the correlation func-tion, which is independent of k^2 but varies with temporal lag. Its saturation value vs τ could be useful if it was not for the noise in the data. Indeed, the r_{inf} is recovered from the very large range of k^2 where the background noise affects mostly the kICS CF. Therefore, this parameter is not used for the characterization of experimental data, but is extracted from the simulation data of the present work. We chose to fit the correlation function with the sum of exponentials vs k^2 at a given τ because there is only a small range of useful temporal lags (~200 statistically significant time lags for image series of 2000 frames) while there are over ~1200 of k^2 values in a statistically sig-nificant range.

Extraction of domain size and particles mobilities

In order to extract characteristic system parameters such as domain size and particles mobilities, from $D\tau$ vs τ plots, we employ the weighted linear least squares fit. The weight used in this fit is the inverse of the square of the error bars (defined by the 95 % confidence intervals) of each $D\tau$ point obtained in the previously described fits. In other words, the following parameter is minimized:

$$s = \sum_{i=1}^{n} \frac{(D\tau|_{exp}^{i} - D\tau|_{fit}^{i})^2}{\sigma_{D\tau}^2} \tag{S3}$$

where $\sigma_{D\tau}$ represents the 95% confidence i nterval o n $D\tau|_{exp}^{i}$ r ecovered from the fit vs k^2 as explained a bove. Therefore, if the parameter $D\tau|_{exp}^{i}$, extracted at a given τ, has a high uncertainty (i.e. larger than $\sigma_{D\tau}$), then the weighted least squares fit will give less weight to that value in the fit.

In order to extract the domain size, the micro component decay $D\tau|_\mu$ was plotted vs the temporal lag variable τ and the saturation point of the curve gives a value that is proportional to the radius of the domain squared. The saturation point is extracted by fitting t he w eighted l inear regression, through the later temporal lags (typically the second half of the temporal lags). Figure 4 a) shows an example of a domain size extraction by weighted linear least squares fit for an isolated domains simulation. Similarly, D_μ is

extracted by applying the weighted linear least square fit to the first 10 tem-poral lags (Figure S4b). Finally, D_M is recovered in a similar fashion (Figure S4c), reflecting the effective diffusion coefficient of particles at large spatial scales.

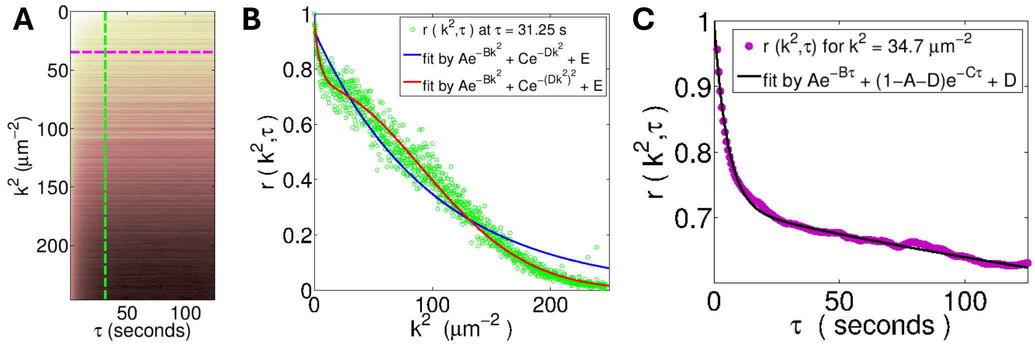

Figure S3: Non-linear least squares fitting k-space correlation function result-ing from a heterogeneous environment. (**A**) 2D magnitude plot of r(k^2, τ) calculated from simulated images. (**B**) Plot of green dashed line from (**A**) with two possible implementations of 2 Gaussian fit. (**C**) P lot o f m agenta dashed line from image on A) with its 2 Gaussian fit.

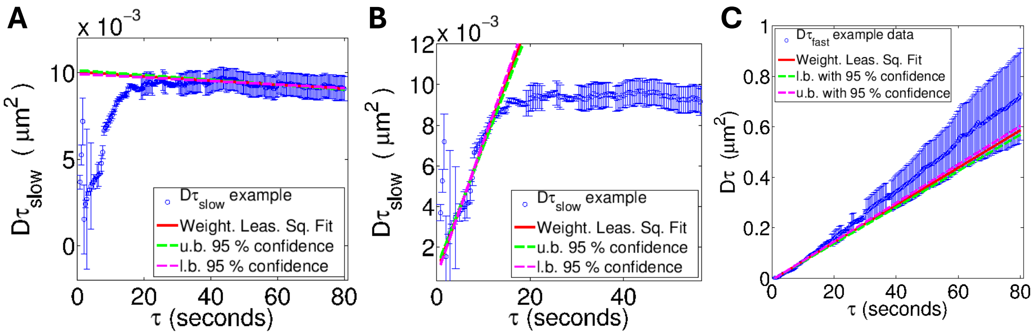

Figure S4: Example of data processing to extract domain size and particle mobilities. (**A**) The blue symbols are micro component mobilities Dτ with 95% confidence intervals. Red line shows weighted least square linear fit and dashed green and magenta lines show 95 % confidence intervals on fitted line. (**B**) Same description as for left example, but for early slope of micro component. (**C**) Same descriptions but for slope of macro component.

References

[1] J.A. Schwartzentruber, *k-space Image Correlation Spectroscopy: accu-racy and precision, capabilities and limitations*, Master Thesis, McGill University, 2010.

[2] D.L. Kolin, *k-space Image Correlation Spectroscopy: theory, verification, and applications*, PhD Thesis, McGill University, 2008.

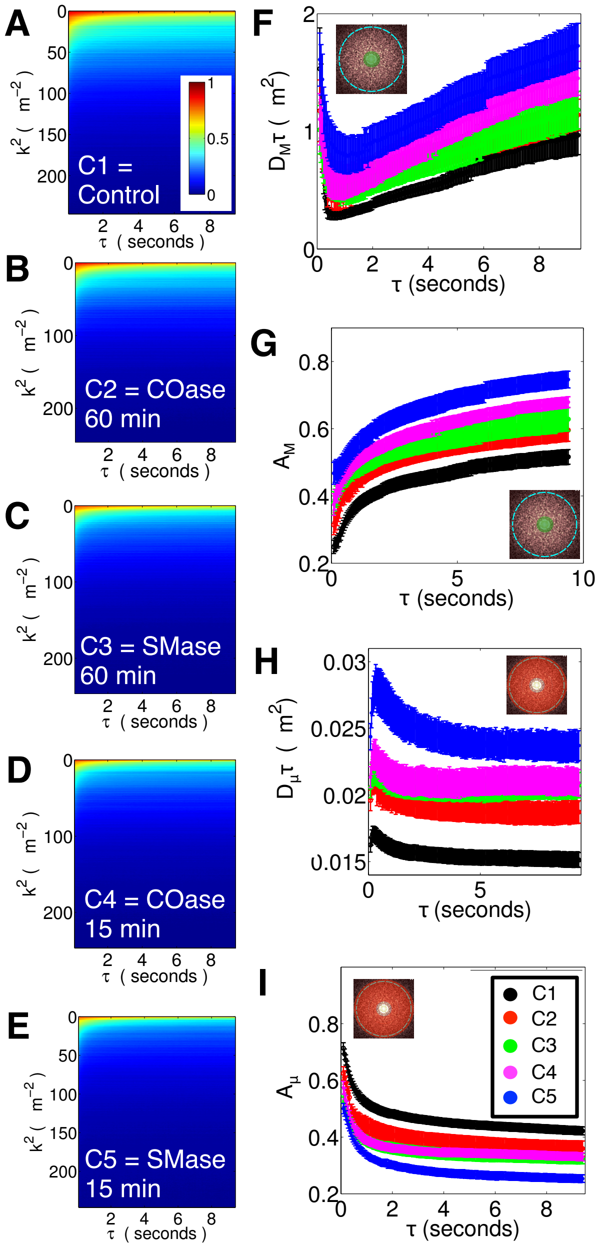

Figure S5: (**A**-**E**) Examples of an average correlation functions for GPI-GFP-antiGFP-Alexa-594 data for 5 enzymatic conditions considered. (**F**,**G**) Fitted parameters as a function of time lag for the two characteristic macro and micro components. Each trend is an average and standard deviation for 20 cells for a given enzymatic treatment. Symbols colors in (**F**-**H**) are same as in the legend of (**I**).

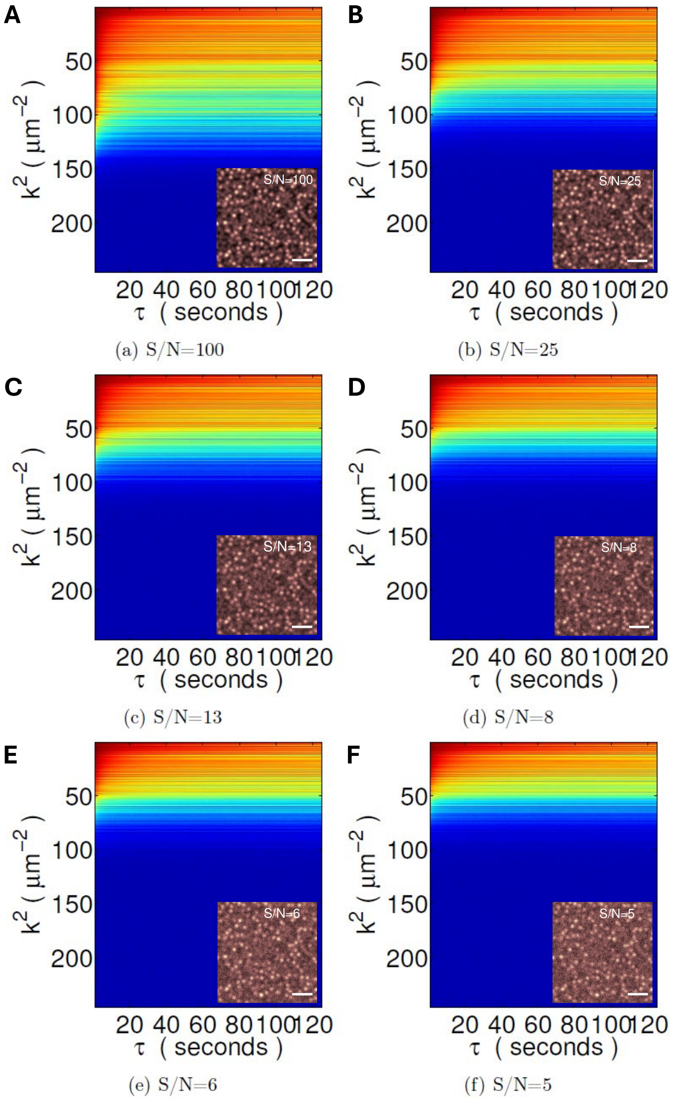

Figure S6: Examples of c-kICS correlation functions emerging from simulated confinement case, with varying background noise in image data. Signal to noise ratio was simulated at ratio of (**A**) 100 (**B**) 25 (**C**) 13 (**D**) 8 (**E**) 6 and (**F**) 5. Each kICS correlation functions also contains an inset showing a snapshot of simulated images with noise added at defined level, bright spots showcasing locations of membrane domains. Scale bar is 5 μm.

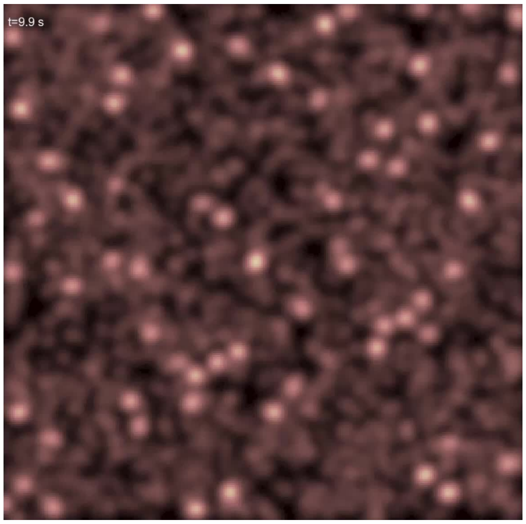

Movie S1 (cover image): Time-lapse of simulated data with P_{in} =0.2 while P_{out} =0.1. The frame rate was set to 0.9 s. Other simulation parameters are default parameters used for simulations and are summarised in Table 1. The video shows 100 frames of simulated data set, where prob-ability of molecular partitioning into domains is only twice as high as their probability of escaping domains.

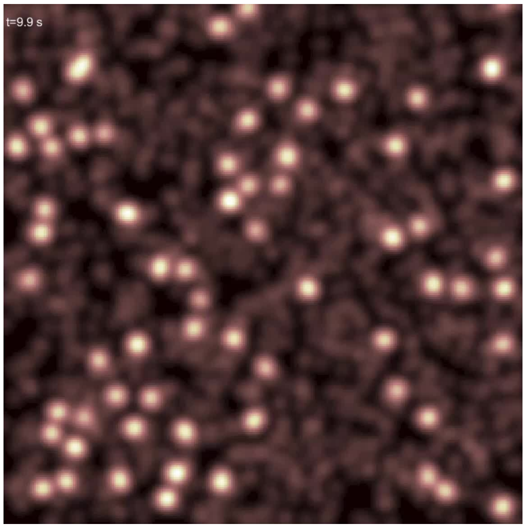

Movie S2 (cover image): Time-lapse of simulated data with P_{in} =0.5 while P_{out} =0.1. The frame rate was set to 0.9 s. Other simulation parameters are default parameters used for simulations and are summarised in Table 1. The video shows 100 frames of simulated data set, where prob-ability of molecular partitioning into domains 5 times their probability of escaping domains, hence why we see higher confinement than in the Supp. Video. 7

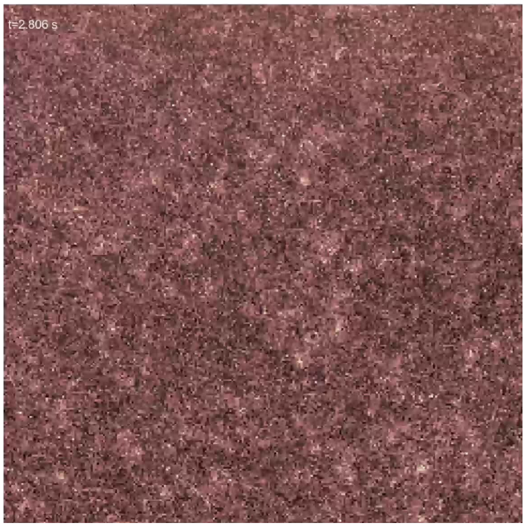

Movie S3 (cover image): Example of a control (no drug treatement) data set of GPI-GFP labeled by Anti-GFP-Alexa555 in COS-7 membranes, imaged in TIRF mode at 46 ms per frame.

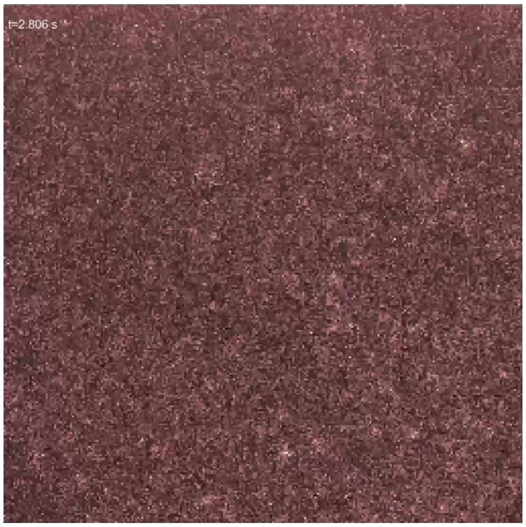

Movie S4 (cover image): Example of a Coase treated sample of GPI-GFP labeled by Anti-GFP-Alexa555 in COS-7 membranes, imaged in TIRF mode at 46 ms per frame.

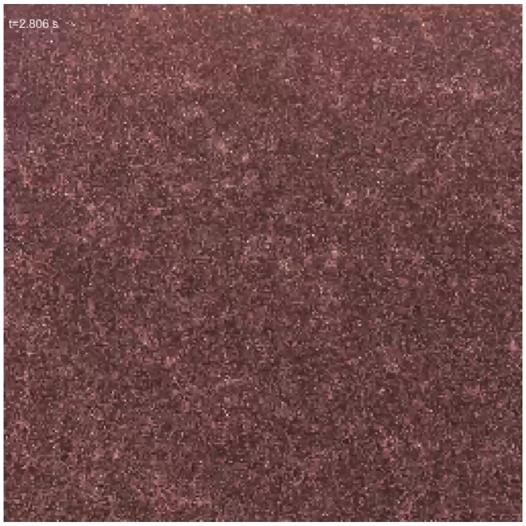

Movie S5 (cover image): Example of a Smase treated sample of GPI-GFP labeled by Anti-GFP-Alexa555 in COS-7 membranes, imaged in TIRF mode at 46 ms per frame.

Table S1: Adjustable parameters for isolated domains simulations

Parameter name	Default values
Image Series Properties	
Number of pixels in x dimension	300 cropped to 256
Number of pixels in y dimension	300 cropped to 256
Number of frames	2200 used last 2000
Pixel size	0.1 μm
t_{frame}: Time between frames	$\frac{(\frac{\text{domain radius}}{5})^2}{4D_{in}}$
Imaging mechanism	CCD-type integration
PSF type	2D Gaussian
$\omega_{x,y} = \omega_0$: PSF e^{-2} x, y radius	0.28 μm
PSF e^{-2} z radius	0 (i.e. 2D simulations)
Particle properties	
Particle density	5 per μm^2
Particle distribution (initially)	uniformly random
Photophysics considered	none
Quantum yield	1
D_{in}: diffusion coefficient inside domains	0.001 to 0.01 $\frac{\mu m^2}{s}$
D_{out}: diffusion coefficient outside domains	0.01 $\frac{\mu m^2}{s}$
Noise	
Background Noise (inverse $\frac{S}{B}$)	0 to 0.2
Domain properties	
P_{in}: probability for particles to enter domains	0.1 to 0.9
P_{out}: probability for particles to exit domains	0.1 to 0.9
r_{domain} : domain radius	0.05 to 0.5 μm
η_d: domain area fraction (% total area)	0.5 to 5 %
domain distribution	uniformly random
domain motion mode	none
